# Miniaturization of mechanical actuators in skin-integrated electronics for haptic interfaces

**DOI:** 10.1038/s41378-021-00301-x

**Published:** 2021-10-22

**Authors:** Dengfeng Li, Jiahui He, Zhen Song, Kuanming Yao, Mengge Wu, Haoran Fu, Yiming Liu, Zhan Gao, Jingkun Zhou, Lei Wei, Zhengyou Zhang, Yuan Dai, Zhaoqian Xie, Xinge Yu

**Affiliations:** 1 Department of Biomedical Engineering, City University of Hong Kong, Hong Kong SAR, China; 2 Hong Kong Centre for Cerebro-Cardiovascular Health Engineering (COCHE), Hong Kong SAR, China; 3 State Key Laboratory of Structural Analysis for Industrial Equipment, Department of Engineering Mechanics, Dalian University of Technology, Dalian, China; 4 Institute of Flexible Electronic Technology of Tsinghua, Jiaxing, China; 5 Tencent Robotics X, Shenzhen, China; 6 Ningbo Institute of Dalian University of Technology, Ningbo, China

**Keywords:** Electrical and electronic engineering, Electronic properties and materials

## Abstract

Skin-integrated electronics, also known as electronic skin (e-skin), are rapidly developing and are gradually being adopted in biomedical fields as well as in our daily lives. E-skin capable of providing sensitive and high-resolution tactile sensations and haptic feedback to the human body would open a new e-skin paradigm for closed-loop human–machine interfaces. Here, we report a class of materials and mechanical designs for the miniaturization of mechanical actuators and strategies for their integration into thin, soft e-skin for haptic interfaces. The mechanical actuators exhibit small dimensions of 5 mm diameter and 1.45 mm thickness and work in an electromagnetically driven vibrotactile mode with resonance frequency overlapping the most sensitive frequency of human skin. Nine mini actuators can be integrated simultaneously in a small area of 2 cm × 2 cm to form a 3 × 3 haptic feedback array, which is small and compact enough to mount on a thumb tip. Furthermore, the thin, soft haptic interface exhibits good mechanical properties that work properly during stretching, bending, and twisting and therefore can conformally fit onto various parts of the human body to afford programmable tactile enhancement and Braille recognition with an accuracy rate over 85%.

## Introduction

Electronic skin (e-skin) is a novel wearable device conformally mounted on the skin with multiple sensing capabilities and is gradually showing advantages in the fields of biomedical engineering, healthcare monitoring, human–machine interactions, etc^1–5^. Benefiting from its good stretchability, skin compatibility and excellent responsiveness to pressure, strain, and temperature, e-skin tends to mimic human skin by sensing external stimuli^6–8^. Based on wearable tactile sensors, such as transistor-based strain sensors^9–11^, resistive sensors^12,13^, and nanogenerators^14–19^, the corresponding applied force and temperature can be measured to provide tactile information. When a prosthesis contains e-skin with many sensors, the acquired tactile signals can be transmitted to the artificial nervous system to provide the prosthesis with tactile sensation, corresponding exactly to the skin’s ability to perceive passive sensations^20–23^.

The active sensation is also gradually expanding the applications of e-skin and bringing new innovations. Based on various stimulation modes, including electrical stimulation and mechanical vibration, scientists have developed actively stimulated haptic interfaces^24–27^. Skin is the largest organ of the human body in the sensory system, and nerves are capable of transmitting physical stimuli to our brain so we can feel tactile sensations. Based on these natural properties of the nervous system, human–machine interactions have been created for various applications. For instance, in prosthetic control and sensing, passive e-skin on the prosthesis acquires tactile signals and then sends commands to control and actuate feedback via active e-skin, thus allowing amputees to feel “touch” again^28,29^. We have recently reported applications associated with skin virtual and augmented reality (VR/AR) by developing soft mechanical actuators as haptic interfaces and providing the possibility of feeling touches and hugs from friends through video chats^24^. To realize haptic reproduction in VR/AR, it is essential to develop an active e-skin that can receive multiple controllable active haptic stimulations on the skin. For example, attaching a haptic interface with actuators onto the hand or finger to recreate a VR touch will be interesting, and it will seem as if one had actually touched an object. Moreover, in active e-skin, haptic feedback with mini actuators operation with high resolution is extremely important, as they offer more types of haptic stimulations ranging from intensities to areas to patterns. However, integrating an actuator array onto a finger to provide tactile patterns is still challenging, since the width of the finger is often <2 cm. Therefore, reducing the size of the actuator and integrating more actuators per unit area is essential.

In this paper, we report a class of materials and mechanical designs for the miniaturization of mechanical actuators and the integration of mini actuators into e-skin-based haptic interfaces. The mechanical actuator uses an electromagnetically driven method and works in vibrotactile mode while exhibiting dimensions of 5 mm diameter and 1.45 mm thickness, which can be successfully integrated into a 3 × 3 array in a 2 cm × 2 cm area to offer programmable haptic feedback on a fingertip. The mini actuator has a resonance frequency near that of skin and is most sensitive to a frequency of 200 Hz, and it exhibits strong intensity vibrations with amplitudes of 1.55 mm at a 0.5 V input. The 3 × 3 array-based haptic interface offers multimodal subregional stimulations in a small area, which could significantly narrow the resolution gap between passive and active e-skin, thereby allowing the corresponding haptic sensations to be transmitted and expressed between these two different types of e-skins. The e-skin haptic interface also demonstrates good performance in Braille recognition, which will greatly facilitate communication among sightless people by delivering traditional Braille to them in a VR/AR-motivated way. The designs of the vibrotactile mini actuators and high-resolution arrays will extend the applicability of AR e-skins.

## Materials and methods

### Fabrication of the mini actuators

The mini actuator involves four parts: a Cu coil, a 3D-printed polymer ring, a magnet, and a laser-cut polyimide (PI) film (Supplementary Fig. S1a). The Cu coil was fabricated with 50 turns of enameled copper wires with a diameter of 0.05 mm to form a concentric disc coil with an inner diameter of 2 mm, an outer diameter of 5 mm, and a thickness of 0.25 mm (Yisu Electronics, Inc., Dongguan China). The supporting polymer ring with an inner diameter of 4 mm, an outer diameter of 5 mm, and a thickness of 1 mm was printed with a photopolymer (General Purpose Transparent RGD720, Stratasys) using an Eden 260 Polyjet system (Stratasys) with a resolution of 600 dpi. With laser-cutting equipment, the 40 µm-thick PI film was cut into thin PI discs with different central angles ranging from 30° to 180° (Fig. 2a, f). To fabricate the mini actuator, the support polymer ring was first bonded on the Cu coil with UV-curable glue, and then a 0.5-mm-thick magnet with a diameter of 2 mm was bonded onto the cut PI disc. After sticking the PI film on the support ring with the magnet facing down, the actuator was acquired. The design and dimensions of the actuator are shown in Supplementary Fig. S2.

### Fabrication of the e-skin

The entire fabrication process of the e-skin haptic interface is summarized in Supplementary Fig. S1. First, a stretchable Cu pattern served as the flexible electrode for the e-skin. A 6-µm-thick copper sheet was flattened on a cured polydimethylsiloxane layer (PDMS, pre-polymer and crosslink agent = 30:1, Sylgard 184, Dow-Corning) on the glass substrate and then coated by positive photoresist (AZ 5214) at 3000 rpm for 30 s with a soft bake at 110 °C for 3 min^30^. Through the processes of UV exposure with a designed film mask, development in the developer (AZ 300 MIF), and wet etching in a FeCl_3_ solution, the designed Cu pattern was acquired. The next step involved integrating and assembling the flexible electrode and the mini actuators. With water-soluble tape (WST), the flexible Cu electrode was transferred and bonded onto a 0.3-mm-thick PDMS (pre-polymer and crosslink agent = 10:1) substrate called silicone A. With low-temperature solder adhesive, the actuators were electrically connected on the Cu electrode to form a 3 × 3 array. Then, the anisotropic conductive film (ACF) wire was connected to a Cu electrode for further actuation by an external current source. Finally, PDMS mixed with white pigment (silicone B, PDMS, pre-polymer and crosslink agent = 30:1) was poured onto the silicone A substrate and Cu electrode with a 2-mm-thick mold. The thickness of PDMS was controlled to the same height as the actuators to avoid silicone covering or filling into the actuator cavity. After cutting with blades, the encapsulated e-skin was acquired with mini actuators with a thickness of ~1.8 mm.

### Characterization and measurement

For basic research on resonant frequencies and amplitudes of actuators with different central angles, their actuations were supplied by an AC current source (Model 6221, Keithley Instruments, Inc.) with certain sinewave peak currents (1.12–56.18 mA, 0.01–0.5 V) and frequencies (70–740 Hz). The vibrations of actuators were recorded by a high-speed camera system from Keyence Corporation of America with 1000 fps, which contributed to determining the quantitative frequencies and amplitudes for all actuators. To test the actuation and control of specific array patterns, an e-skin system was connected to a designed printed circuit board by an anisotropic conductive film (ACF) wire and then connected with a multi-circuit relay that could be controlled through a computer software interface. The detailed circuit diagram connections are shown in Supplementary Fig. S5. All actuators were connected in series, and the operation of each actuator was controlled by a short circuit at both ends of the actuator. When the actuator is short-circuited, the actuator is off, and conversely, the corresponding actuator works. The short-circuit switch was controlled by the multi-circuit relay. All movies were recorded with a macrolens camera (SONY).

### Finite element analysis (FEA)

The commercial software ABAQUS was used to study the mechanical performances of the devices. The layouts of each layer and the shapes of the interconnects were optimized to decrease strain/stress levels in the interconnects caused by typical external loads (stretching, bending, and twisting). The resonance frequency of the actuator can be tuned by designing the central angle *θ* of the PI layer in the actuator to increase the vibration intensity of the magnets. The magnet, PDMS, and polymer ring were modeled by hexahedron elements (C3D8R), while the thin copper and PI film were modeled by composite shell elements (S4R). The minimum element size was 1/4 of the width of the interconnects (100 µm). The mesh convergence of the simulation was guaranteed for all cases. The elastic moduli (*E*), Poisson’s ratios (*ν*), and densities (*ρ*) are shown in Table 1.

**Table 1:** Mechanical parameters of the components of actuators and e-skin for finite element analysis.

Components/parameters	Elastic modulus (*E*)	Poisson’s ratio (*ν*)	Density (*ρ*)
Cu (flexible electrodes)	119 GPa	0.32	8.96 × 10^3^ kg/m^3^
Magnet	113 GPa	0.34	8.08 × 10^3^ kg/m^3^
PI	4.0 GPa	0.34	0.91 × 10^3^ kg/m^3^
Silicone A	1 MPa	0.5	0.96 × 10^3^ kg/m^3^
Silicone B	145 kPa	0.5	0.96 × 10^3^ kg/m^3^
Ring	300 MPa	0.45	1.2 × 10^3^ kg/m^3^

## Results and discussion

**Fig. 1:**
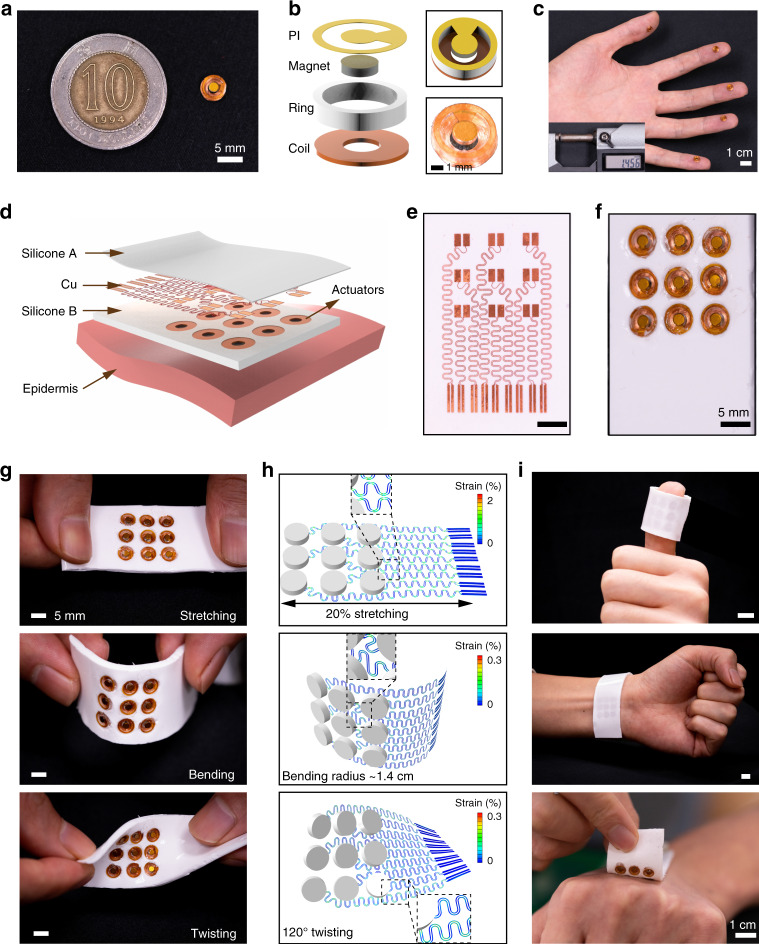
Design and presentation of the mini vibrotactile actuators and e-skin. **a** Photograph of a ten-dollar Hong Kong coin and a 5 mm vibrotactile actuator. **b** Exploded-view and top-view schematic diagram of a mini actuator. **c** Photograph of actuators on fingers and thickness display in the inset picture. **d** Exploded-view schematic illustration of the e-skin with 9 independently controlled mini actuators. **e** Optical images of the flexible Cu electrode. **f** Optical images of the e-skin with a view from above. **g**, **h** Optical images (left column) and FEA results (right column) of the e-skin under stretching, bending, and twisting. **i** Optical images of the e-skin mounted on the finger, wrist, and hand back

Figure 1 shows the designs of the mini vibrotactile actuator and the e-skin haptic interface. The diameter of the actuator is only 5 mm, which is much smaller than a ten-dollar Hong Kong coin (Fig. 1a). Figure 1b shows the schematic diagram and an optical image of the actuator, in which the device consists of a copper (Cu) coil serving as a magnetic field generator, a polymer ring acting as the backbone, a magnet serving as the vibrator, and a thin polyimide (PI) film holding the magnet. Enameled Cu wires with a diameter of 50 m were wound into 50 turns to form Cu coils, whose parameters are 2 mm inner diameter, 5 mm outer diameter, 0.05 mm thickness, and resistance of 8.9 Ω. A high-resolution 3D-printed polymer ring (4-mm inner diameter, 5-mm outer diameter, 1-mm thickness) served as the shell to provide free space for the magnet to vibrate. A 0.5-mm-thick magnet disc with a diameter of 2 mm was bonded on a PI film and attached to the ring with the magnet pointing down in the supporting ring. To tune the resonance frequency for the actuator to provide more intense haptic feedback, a 40-µm-thick PI supporting film was designed and cut into PI discs with different central angles by using a laser-cutting machine. The precise dimensional parameters of each component in the actuator are shown in Supplementary Fig. S2. After assembling all parts together with UV-curable glue, the overall dimensions of the mini actuator were 5 mm in diameter and 1.45 mm in thickness (Fig. 1b, c). The mini actuators are small enough to be used on a small area of any finder tip (Fig. 1c). Nine vibrotactile mini actuators can be put together in a small area to form a 3 × 3 actuation array via a connection on a preformed stretchable Cu electrode and then encapsulation with a soft silicone polymer (Fig. 1d). The integration of the array starts on a stretchable Cu circuit prepared with a 6-µm-thick copper sheet by methods described in our previous works^31–35^. Through photoresist spin-coating, soft-baking, UV exposure, development, and wet etching, a patterned Cu electrode was acquired. Then, transfer of the Cu electrode onto 0.3-mm-thick polydimethylsiloxane (PDMS, silicone A) substrate (pre-polymer and crosslink agent = 10:1) formed the flexible circuit (Fig. 1e). Low-temperature soldering of these mini actuators onto Cu electrode patches with a 2 cm × 2 cm area and then bonding with an anisotropic conductive film (ACF) wire enabled connection with an external power supply. Finally, PDMS mixed with white pigment (silicone B, PDMS, pre-polymer, and crosslink agent = 30:1) encapsulated the circuit and the actuator array. The thickness of the encapsulation PDMS was controlled with a mold to the same height as the actuators. The final e-skin interface with a 9 vibrotactile actuator array (1.8 mm thick) is shown in Fig. 1f. Advanced mechanical designs guided by finite element analysis (FEA) provided an e-skin interface with stretchable electrodes and excellent mechanical performance under typical physical deformations (Fig. 1g), including stretching, twisting, and bending. Figure 1h summarizes the FEA results corresponding to each type of deformation. With 20% uniaxial stretching, the maximum equivalent strain in the Cu layer was significantly <5% of the fracture strain of copper, while for a 1.4 cm bending radius and a 120° twisting angle, the maximum equivalent strain in the Cu layer was below the 0.3% yield strain, making the deformation reversible. The excellent mechanical properties of the e-skin interface combined with the soft and thin design allow for conformal operation on fingers, wrist, and back of the hand (Fig. 1i and Supplementary Fig. S3).

**Fig. 2:**
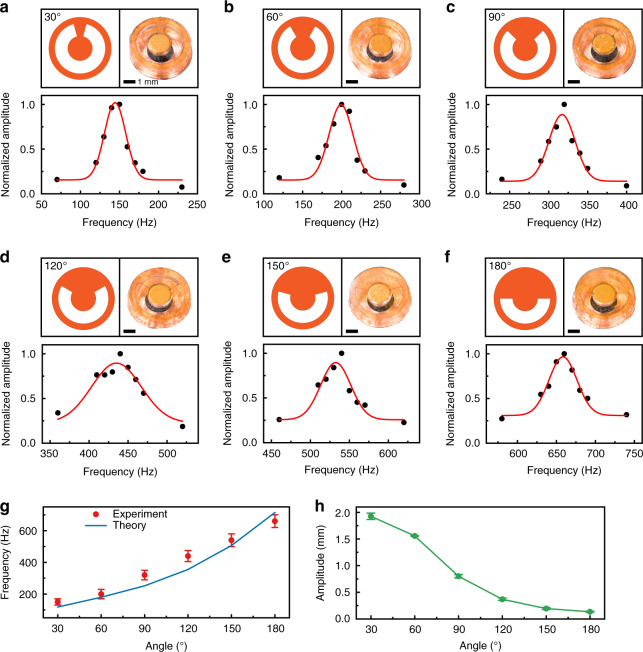
Optimization of frequency and amplitude of the vibrotactile mini actuators. **a–f** PI discs with different central angles ranging from 30° to 180° (top row, left), top views of the corresponding actuators (top row, right), and normalized amplitude–frequency responses of actuators (bottom row). **g** Dependence of the resonance frequency on the central angle. **h** Amplitude changes of the actuators at the resonance frequency with the central angle

Due to the intrinsic nature of mechanoreceptors under the skin, such as Meissner’s corpuscles and Pacinian corpuscles with low thresholds of activation and rapid rates of adaptation, the most sensitive tactile sensations we feel are associated with vibration frequencies ranging from 100 to 300 Hz^36–38^. Therefore, developing high-efficiency vibrotactile actuators operating at 100–300 Hz is critical for developing high-performance haptic interfaces. Figure 2 shows the optimizations of the actuators that involve studying their frequencies and amplitudes as functions of the specific design parameters. Based on the designs of materials and structures, the resonance frequency of the mini actuator can be adjusted to be ~200 Hz, which is in the middle of the most sensitive frequency range. Formation of the PI film into a cantilever-like platform with beams at different central angles was the design strategy used to allow the magnets to exhibit different vibration behaviors in the cavity. The corresponding resonance frequencies can be tuned precisely by changing the central angles. The top rows in Fig. 2a–f show the designs of the PI disc with different central angles ranging from 30° to 180° and top-view photos of the corresponding assembled actuators. Under input of sinusoidal alternating current (AC) with a peak value of 56.18 mA, the vibrations of these actuators were recorded by a high-speed camera system (Supplementary Movie S1). Fitting the normalized amplitudes with the frequency, the resonance frequencies were obtained from the positions of the peaks (Fig. 2a–f, bottom rows). As shown in Supplementary Movie S2, the vibration amplitude of the actuator with a central angle of 60° reached its peak value at 200 Hz, indicating that the resonance frequency of this device is 200 Hz. For actuators with central angles of 30°, 60°, 90°, 120°, 150°, and 180°, the resulting resonance frequencies were 150, 200, 320, 440, 540, and 660 Hz, respectively. As the central angle decreased, the width of the PI beam decreased, and the magnet was more likely to vibrate at low frequencies in the alternating magnetic field from the coil. The resonance frequencies were consistent with results from FEA simulations (Fig. 2g), demonstrating the great processing accuracy and the successful design strategy. In general, the resonant frequency *f* relates to the effective stiffness *K* and effective mass *M* of a vibrational system as in *f* = (*K*/*M*)^0.5^, where *K* and *M* are dependent on the shape, material properties, and vibrational model of the structure. Therefore, the resonant frequency *f* and angle *θ* do not have a simple linear relationship since *K* and *M* (no change) in our system mainly depend on *θ* and the magnet mass, respectively. In addition to the tuning of the resonance frequency, actuators with small central angles tend to produce higher amplitudes, as shown in Fig. 2h and Supplementary Fig. S4. The actuator with a central angle of 60° has a resonance frequency of 200 Hz and amplitude of 1.55 mm at 56.18 mA and thus was selected as the designated device for the e-skin haptic interface.

**Fig. 3:**
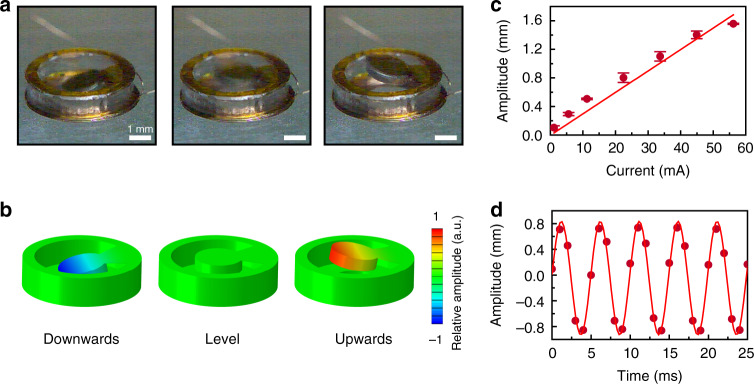
Vibration behaviors of actuators with a resonance frequency of 200 Hz. **a**, **b** Optical images captured using a high-speed camera (**a**) and FEA results (**b**) of the actuator traveling in a downward direction (left columns), leveled, and traveling in an upward direction. **c** Relationship between actuator amplitude and input peak sinewave current. **d** Values of the vibration amplitude with time

For the actuator with a central angle of 60°, the vibration behaviors are summarized in Fig. 3. Figure 3a shows high-speed camera images capturing different positions of the actuator, including traveling downwards, natural state, and traveling upwards. The corresponding FEA simulation results for these three working statuses are shown in Fig. 2b and Supplementary Movie S4. The direction of magnetism for the 0.5-mm-thick magnet was out of plane (upward direction). Therefore, the direction of the magnetic field induction lines inside the magnet was the same as that generated by the coil when an external current was supplied, and therefore, the magnet moved in the magnetic direction of the coil. When the magnetic direction of the coil was switched to the opposite direction, the magnet also changed its moving direction simultaneously, which resulted in periodic vibration, as shown in Fig. 3d. The amplitude distribution as a function of time exhibited the same sinusoidal-type distribution as the input current signal. Moreover, the peak-to-peak amplitude of the 60° actuator at 200 Hz frequency showed an approximately linear relationship with the value of the input current (Fig. 3c). As shown in Supplementary Movie S3, the amplitude of the actuator at 200 Hz reached 0.28 mm with an extremely small input power input of 1.4 mW (0.05 V, 5.62 mA), which provides skin with a very obvious tactile feeling since the sensation threshold of vibration in human skin is as low as several micrometers^38^. A 200 Hz AC peak current with a value of 56.18 mA can induce an amplitude as high as 1.55 mm without significant heat generation since the power supplied by the actuator is only 14 mW due to the extremely low resistance of the device.

**Fig. 4:**
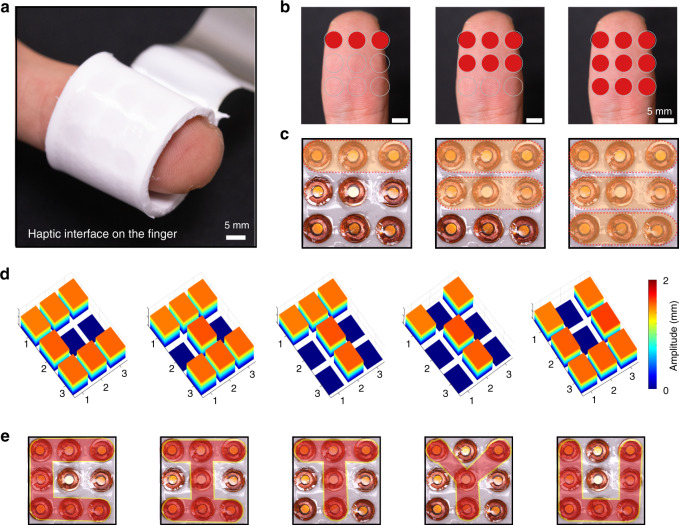
Programmable control and pattern actuation of e-skin for a haptic interface. **a** Photograph of e-skin on a finger. **b**, **c** Subregional control of the e-skin. Optical images of the corresponding finger area (**b**) and actuated area of the array (**c**). **d**, **e** Programmable actuation of the e-skin in a “CITYU” pattern. Amplitude distributions (**d**) and optical images of the programmed actuation area (**e**)

Based on the alternating electromagnetic fields generated by these mini actuators, the mechanical forces of the magnets allow the skin to experience haptic reproduction, which is different from the strain sensors that generally act as detectors of the force applied to the skin. Using stretchable electrodes, silicone encapsulation, and nine actuators with central angles of 60°, the system can be integrated into e-skin with a 3 × 3 actuation array for use as a haptic interface on a fingertip (Fig. 4a). When connected with a multi-circuit relay as in the circuit diagram in Supplementary Fig. S5, the e-skin exhibited programmable control and pattern actuation. A multi-circuit relay is the functional equivalent of a series of switches that can be controlled independently. All actuators were connected in series, and each actuator was individually shorted with the multi-circuit relay in its initial nonworking state. Under the control of the multi-circuit relay with a LabVIEW visual interface in the computer, the activated actuators worked when the corresponding short-circuit switch was on. The power was supplied from a constant AC current source, and the operating state of each actuator was uniform since all actuators were connected in series. The integration of 9 actuators at the fingertip of the thumb allowed for zoned control. Figure 4b, c shows the stimulation area of the actuators and the area corresponding to the fingertip, including single-row, double-row, and full-area actuation. Additionally, the effective control also allowed the actuation of a complex pattern. Here, we show programmed actuation for a pattern of “CITYU” with the amplitude distribution and actuation area shown in Fig. 4d, e. All actuation and control processes were recorded with a camera, as shown in Supplementary Movie S5.

**Fig. 5:**
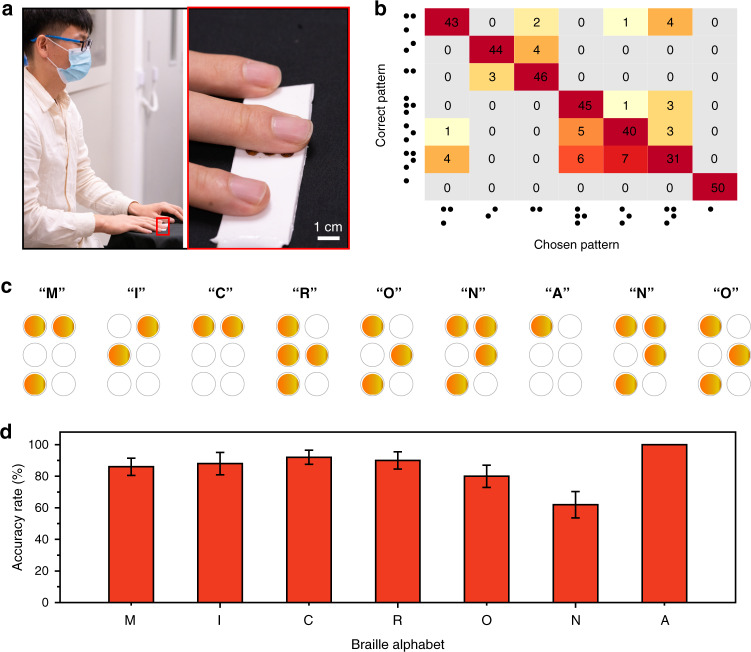
Braille recognition based on the haptic interface. **a** Photograph of the tester’s finger placed on the haptic interface. **b** Grid view of the test results. **c** Braille letters “MICRO-NANO” and the corresponding actuator positions. **d** Testing accuracy rate for braille letters “M, I, C, R, O, N, A”

To further demonstrate the power of the e-skin haptic interfaces with these miniaturized actuators, we took the case of Braille recognition as an example. Figure 5a shows that a tester placed his finger on this haptic interface to feel the vibrations of the actuators and further identify the applied pattern. In this test, a point-by-point sensing method was used to perform Braille pattern recognition. The letters used were “MICRO-NANO”, and the distribution of the corresponding patterns is shown in Fig. 5c. Since there are similarities among the patterns, some patterns are likely to be confused with other, similar patterns. For each letter, five testers were involved in ten groups of blind tests, totaling 50 sets of data. During the test, the tester did not know the real pattern in advance. They needed to feel the pattern with their fingertips and then write the pattern sensed, called the chosen pattern. The actuation process of the letters was recorded in Supplementary Movie S6. As shown in Fig. 5b, a grid map was used to summarize the correlation between the chosen pattern and the correct pattern during testing. Misidentification mainly occurred among letters within three sets, “RON”, “IC”, and “MN”. The simpler the array was, the higher the accuracy of recognition. For “N” pattern recognition, the accuracy was only 62%. For the letter “A” with only a single actuator working, the accuracy reached 100%. For these seven letters, the average recognition accuracy was 85.4% (Fig. 5d). These test results all show that this haptic interface is sufficient for Braille recognition based on sensing by fingertip areas and will provide a great convenience for mutual communication among sightless people.

In the future, this haptic interface could also be used in VR and AR applications involving direct human-to-human tactile communication. Such high resolution will make the haptic sensation more realistic. Furthermore, the introduction of new high-precision fabrication processes and mechanical designs is also possible for further reducing the sizes of actuators and significantly improving the resolution of the haptic interfaces, allowing it to match the tactile spatial resolution of the skin. In the game and entertainment experience, it will also offer gamers more realistic scenarios. With the help of system integration aided by future circuit design, commercially available high-resolution haptic interfaces will be very promising.

## Conclusion

In summary, we reported a comprehensive development strategy for the miniaturization of mechanical actuators for high-resolution haptic interfaces, in which the mechanical actuators can be as small as 5 mm in diameter and 1.45 mm in thickness. Each vibrotactile actuator works at the resonance frequency of 200 Hz, the median value of the vibrational frequency region to which the human body is most sensitive. Its large amplitude with a value of 1.5 mm allows the haptic interface to impart a very strong sensation of vibration to human skin. The haptic interface integrating 9 actuators to achieve a 3 × 3 array with controllable and programmable actuation patterns can be used in very localized areas of skin, such as fingertips. Based on this programmable intense tactile stimulation, this VR haptic interface was applied to Braille recognition and demonstrated a reasonable accuracy rate of 85.4%, which brings considerable convenience to communication by sightless individuals. The development of this mini actuator and the high-resolution haptic system will hopefully lead to a more realistic haptic experience in AR/VR.

## Supplementary information

Supplementary Information

Movie S1

Movie S2

Movie S3

Movie S4

Movie S5

Movie S6
