# Supplementary material for: Miniaturization of mechanical actuators in skin-integrated electronics for haptic interfaces
Source: Microsyst Nanoeng. 2021 Oct 22;7:85. doi: 10.1038/s41378-021-00301-x (PMC8536704; doi:10.1038/s41378-021-00301-x)
Supplement: Supplementary file 1 — Supplementary Information [file 41378_2021_301_MOESM1_ESM.docx]

Supplementary Information for

**Miniaturization of mechanical actuators in skin-integrated electronics for haptic interfaces**

Dengfeng Li^1,2^, Jiahui He^1^, Zhen Song^3^, Kuanming Yao^1^, Mengge Wu^1^, Haoran Fu^4^, Yiming Liu^1^, Zhan Gao^1^, Jingkun Zhou^1,2^, Lei Wei^5^, Zhengyou Zhang^5^, Yuan Dai^5,*^, Zhaoqian Xie^3,6,*^, Xinge Yu^1,2,*^

1 Department of Biomedical Engineering, City University of Hong Kong, Hong Kong SAR 999077, China.

2 Hong Kong Centre for Cerebro-Cardiovascular Health Engineering (COCHE), Hong Kong SAR 999077, China

3 State Key Laboratory of Structural Analysis for Industrial Equipment, Department of Engineering Mechanics, Dalian University of Technology, Dalian 116024, China.

4 Institute of Flexible Electronic Technology of Tsinghua, Jiaxing 314006, China.

5 Tencent Robotics X, Shenzhen 518054, China.

6 Ningbo Institute of Dalian University of Technology, Ningbo, 315016, China.

*Corresponding author. Email:

[jessiedai@tencent.com](mailto:jessiedai@tencent.com) (Y.D.), [zxie@dlut.edu.cn](mailto:zxie@dlut.edu.cn) (Z.X.), [xingeyu@cityu.edu.hk](mailto:xingeyu@cityu.edu.hk) (X.Y.)

These authors contributed equally: Dengfeng Li, Jiahui He, Zhen Song

**Supplementary Movies:**

Movie S1. Vibration at resonant frequency for the actuators with different central angles under 0.5 V voltage (peak-peak sinewave) input.

Movie S2. Vibration at different frequencies for the actuator with 60° central angle under 0.5 V voltage (peak-peak sinewave) input.

Movie S3. Vibration at 200 Hz, different voltage inputs for the actuator with 60° central angle.

Movie S4. FEA simulated vibration for the actuator with 60° central angle.

Movie S5. Pattern actuation and “CITYU” display of the e-skin with the actuators 3×3 array.

Movie S6. Braille actuation and “MICRO-NANO” display for the e-skin.

**Supplementary Figures:**


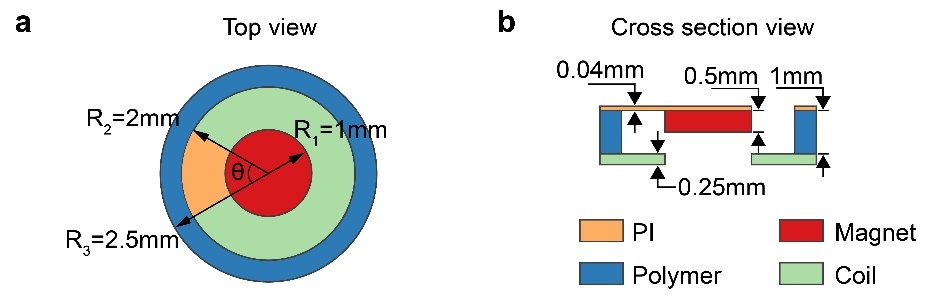


**Fig. S1** Design of the actuator. (a) Top view of the actuator. Central angle of the PI film ranges from 30° to 180°. Diameter of the actuator is 5 mm. (b) Cross-sectional view of the actuator. Designed thickness is 1.29 mm.


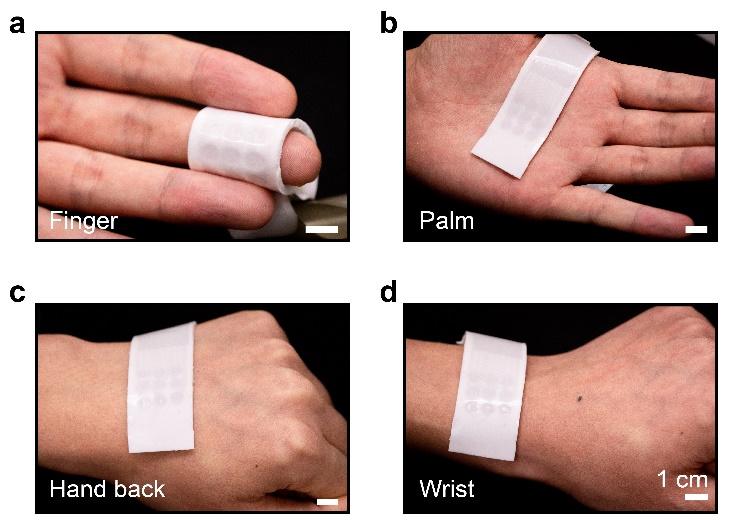


**Fig. S2** The e-skin interface is conformally affixed to the finger (a), palm (b), hand back (c), and wrist (d).


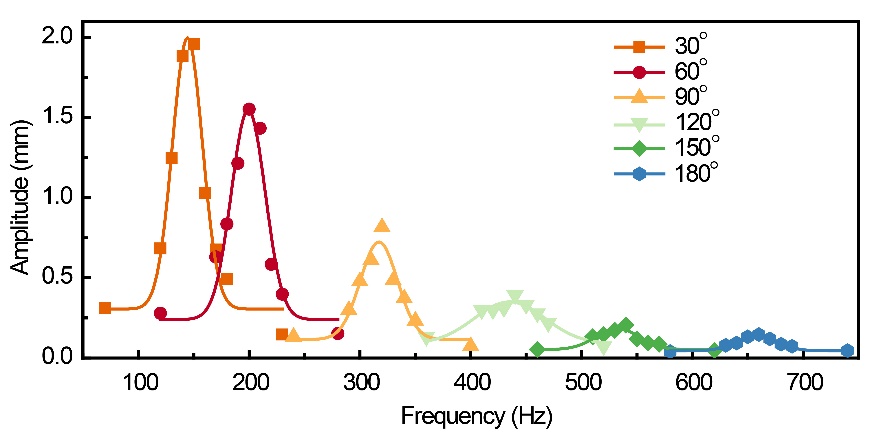


**Fig. S3** Resonant frequency tuning of the actuator. With central angles of the actuators ranging from 30° to 180°, their resonant frequency is tuned from 150 Hz to 660 Hz. The corresponding amplitude of the actuators decreases with the increase of the resonant frequency.


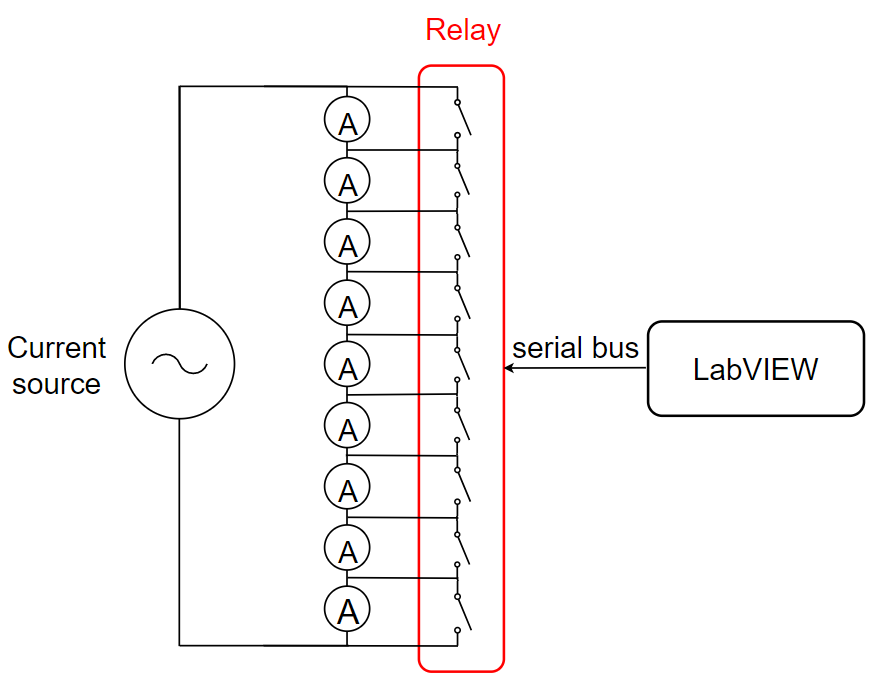


**Fig. S4** The circuit diagram for the actuation and control of the e-skin with 3×3 actuators array.
